# Cysteine Protease Profiles of the Medicinal Plant *Calotropis procera* R. Br. Revealed by *De Novo* Transcriptome Analysis

**DOI:** 10.1371/journal.pone.0119328

**Published:** 2015-03-18

**Authors:** Chang Woo Kwon, Kyung-Min Park, Byoung-Cheorl Kang, Dae-Hyuk Kweon, Myoung-Dong Kim, Sang Woon Shin, Yeon Ho Je, Pahn-Shick Chang

**Affiliations:** 1 Department of Agricultural Biotechnology, Seoul National University, Seoul, Republic of Korea; 2 Department of Plant Science, Plant Genomics and Breeding Institute, and Research Institute of Agriculture and Life Sciences, Seoul National University, Seoul, Republic of Korea; 3 Department of Genetic Engineering and Center for Human Interface Nanotechnology, Sungkyunkwan University, Suwon, Republic of Korea; 4 Department of Food Science and Biotechnology, Kangwon National University, Chuncheon, Republic of Korea; 5 Department of Agricultural Biotechnology, and Research Institute of Agriculture and Life Sciences, Seoul National University, Seoul, Republic of Korea; 6 Center for Food and Bioconvergence, and Research Institute of Agriculture and Life Sciences, Seoul National University, Seoul, Republic of Korea; Tsinghua University, CHINA

## Abstract

*Calotropis procera* R. Br., a traditional medicinal plant in India, is a promising source of commercial proteases, because the cysteine proteases from the plant exhibit high thermo-stability, broad pH optima, and plasma-clotting activity. Though several proteases such as Procerain, Procerain B, CpCp-1, CpCp-2, and CpCp-3 have been isolated and characterized, the information of their transcripts is limited to cDNAs encoding their mature peptides. Due to this limitation, in this study, to determine the cDNA sequences encoding full open reading frame of these cysteine proteases, transcripts were sequenced with an Illumina Hiseq2000 sequencer. A total of 171,253,393 clean reads were assembled into 106,093 contigs with an average length of 1,614 bp and an N50 of 2,703 bp, and 70,797 contigs with an average length of 1,565 bp and N50 of 2,082 bp using Trinity and Velvet-Oases software, respectively. Among these contigs, we found 20 unigenes related to papain-like cysteine proteases by BLASTX analysis against a non-redundant NCBI protein database. Our expression analysis revealed that the cysteine protease contains an N-terminal pro-peptide domain (inhibitor region), which is necessary for correct folding and proteolytic activity. It was evident that expression yields using an inducible T7 expression system in *Escherichia coli* were considerably higher with the pro-peptide domain than without the domain, which could contribute to molecular cloning of the *Calotropis procera* protease as an active form with correct folding.

## Introduction

Proteases are a class of enzymes that occupies a crucial position with respect to physiological roles as well as various industrial and therapeutic applications. Latex from plants has been considered an important source of papain-like cysteine proteases, which are promising candidates with high proteolytic activity and unique characteristics as biocatalysts.

*Calotropis procera* R. Br. (Asclepiadaceae family) is a tropical plant that has been widely used in Indian traditional medicinal system. The latex from this plant has various therapeutic uses, including as hepatoprotective, anti-arthritic, anti-inflammatory, antipyretic, and anticancer treatments [1–5]. Dubey *et al*., and Ramos *et al*. described purification of several cysteine proteases (Procerain, Procerain B, CpCp-1, CpCp-2, and CpCp-3) from the latex of *Calotropis procera* [6–8]. Procerain and Procerain B exhibited high thermo-stability and broad pH optima, which signify commercial importance, and CpCP-1, CpCP-2, and CpCP-3 exhibited plasma-clotting activity mediated by a thrombin-like mechanism. Therefore, it is necessary to obtain proteases in adequate amounts for use in both basic research and industrial applications. However, the preparation of purified enzymes from tropical plant sources depends on several factors, such as environmental conditions for plant growth and the techniques involved in enzyme purification processes which determine activity, purity, and yield.

In order to generate large quantities of desired protein for further study, overexpression system of recombinant proteins in microorganisms was widely adopted [9]. Recombinant proteins can be engineered to be easily purified and increase their stability in extremes of pH and temperature and resistance to autolysis. However, to date, there is no report of expression of the active form of the protein, which may be due to the incomplete complementary DNA (cDNA) sequence, which only encoded mature peptide [10].

All cysteine proteases investigated to date require, for their correct folding, a large pro-region, which can comprise up to 30% of the total molecular weight of the zymogen. This pro-region probably serves a dual function, as both a folding template and an intrinsic inhibitor, preventing ectopic activation of the newly synthesized protein [11–13]. Therefore, the full-length cDNA information encoding full open reading frame (ORF) of enzyme is necessary for efficient expression of the proteases [14].

Next-generation high-throughput RNA sequencing (RNA-Seq) is a recently developed method with several advantages over other expression profiling technologies in terms of robustness, resolution, and inter-laboratory portability [15]. Next-generation sequencing platforms can detect millions of transcripts and can be used for new gene discovery and expression profiling independent of a reference genome [16–18]. In this study, we aimed to identify the complete mRNA sequences of cysteine proteases by RNA-Seq and to determine their expression levels. This is the first report of *Calotropis procera* transcriptome analysis; the cysteine protease profiles obtained provide fundamental information for further molecular cloning.

## Materials and Methods

### Total RNA extraction

Total RNA was isolated from young leaves of *Calotropis procera* using an RNeasy Plant Mini Kit (QIAGEN, Valencia, CA). The tissues were lysed in RLC buffer containing guanidine hydrochloride, and RNA was purified according to the manufacturer’s instructions. Isolated total RNA samples were treated with DNase to remove any contaminating genomic DNA. RNA concentrations were quantified using a NanoDrop spectrophotometer at a wavelength 260 nm. Integrity of the total RNA samples was evaluated using an Agilent 2100 Bioanalyzer (Agilent Technologies, Santa Clara, CA), and samples with RNA integrity values above 8.3 were used in the experiments described below.

### cDNA library construction

cDNA libraries were prepared from total RNA samples using an Illumina TruSeq RNA Sample Prep Kit (Illumina, San Diego, CA). One microgram of total RNA was used as the RNA input according to recommendations of the manufacturer’s protocol. Poly(A) mRNA was purified using oligo(dT)-conjugated magnetic beads and was eluted with Tris-HCl. Fragmentation medium was added to break the mRNA into short fragments. Taking these fragmented mRNAs as templates, random primers and SuperScript II reverse transcriptase (Invitrogen, Carlsbad, CA) were used to synthesize first-strand cDNA. This was followed by second-strand cDNA synthesis using DNA polymerase I and RNaseH. The ends of the resulting double-stranded cDNA molecules were repaired by adding a single ‘A’ base and then Illumina adapters were ligated to the repaired ends. cDNA fragments of 250–350 bp were purified from the gel and subjected to further template enrichment by PCR using two primers that anneal to the ends of the adapters to construct a fragmented cDNA library. The cDNA libraries were validated for RNA integrity and quantity using an Agilent 2100 Bioanalyzer (Agilent Technologies) before sequencing.

### RNA sequencing

The validated cDNA libraries were clustered onto a TruSeq paired-end flow cell and subjected to transcriptome sequencing for 100-bp paired-end reads (2 × 100) using a TruSeq 200 cycle SBS kit (Illumina). After the sequencing platform generated sequencing images, pixel-level raw data collection, image analysis, and base calling were performed using Real Time Analysis (RTA) software (Illumina). The base call files were converted to FASTQ files using the CASAVA v.1.8.0 software (Illumina) for downstream analysis.

### Short read assembly and gene of interest

Raw sequence data were filtered using standard RNA-seq parameters. Briefly, low-quality and N-base reads were trimmed from the raw reads and reads were filtered by Phred quality score (Q≥20 for all bases) and read length (25≥bp). The clean reads were de novo assembled using Trinity (release 20111126) with the fixed default k-mer value of 25, minimum contig length of 200, and paired fragment length of 500 [19]. The clean reads were also assembled using Velvet-Oases (Velvet: version 1.2.10 and Oases: version 0.2.08) [16]. Reads were assembled into contigs at distinct k-mer value (45, 55, 61, 63, 65, 67, 69, and 75) using Velvet. Finally, the contigs were assembled at k-mer values 63 and 65, and merged using Oases with a minimum length of 200 bp and other default settings. Prior to submission of the data to the Transcriptome Shotgun Assembly Sequence Database (TSA), assembled transcripts were blasted to NCBI’s UniVec database (http://www.ncbi.nlm.nih.gov/VecScreen/UniVec.html) to identify segments with adapter contamination and trimmed when significant hits were found. This adapter contamination may result from sequencing into the 3′ ligated adapter of small fragments (<100 bp). Human and bacterial sequence contamination was identified using the web-based version of DeconSeq, with a query coverage and sequence identity threshold of 90%. To find cysteine protease orthologs, we used sequences of Procerain B and CpCP-1 in a local BLAST search (tBLASTn) querying the assembled *Calotropis procera* transcriptome sequences. Hits with an E-value less than 1e-15 were examined. Cysteine protease unigenes were translated over 6 frames using by the ExPASy translate tool (http://web.expasy.org/translate/) and protein functional domains were predicted by using InterProScan 4 web program (http://www.ebi.ac.uk/Tools/pfa/iprscan/).

### Quantitative real-time PCR validation

RT-PCR analysis was conducted to validate the relative expression level of the cysteine proteases. Primer-BLAST was used to check the specificity of the primers listed in S1 Table. The cDNA products derived from mixed samples were used as templates. The expression levels of 15 novel transcripts related to cysteine proteases were determined. The quantitative reactions were performed on an iQ5 real-time PCR detection system (Bio-Rad, Hercules, CA), using SYBR Premix Ex Taq (Perfect Real Time) (Takara Bio, Shiga, Japan). PCR amplification was conducted using the following conditions: 50°C for 2 min and 95°C for 30 sec, followed by 40 cycles of 95°C for 10 sec and 55°C for 25 sec. Expression of cysteine protease was normalized against Ct (threshold cycle) value of internal reference genes, glyceraldehyde-3-phosphate dehydrogenase. Relative expression was calculated by the comparative Ct method (2^-ΔΔCt^) [20].

### Cysteine protease gene cloning

First-strand cDNA was synthesized from 1 μg of total RNA using 1 μL of Quantiscript Reverse Transcriptase (QIAGEN) according to the manufacturer’s protocol. To amplify the SnuCalCp03 gene fragment, an aliquot of first-strand cDNA was used as the template for the synthesis of the second cDNA strand in a PCR reaction, and then subsequent amplification of double-stranded cDNA was performed using a gene-specific forward primer (GSP-F) and a gene-specific reverse primer (GSP-R) (Table 1). PCR amplification was conducted using the following conditions: 98°C for 3 min; 98°C for 10 sec, 55°C for 15 sec, 72°C for 90 sec (27 cycles); 72°C for 10 min. Duplicate PCR products were pooled and purified by gel electrophoresis using a QIAEX II Gel Extraction Kit (QIAGEN). The purified PCR products were cloned using a T-blunt PCR cloning kit (SolGent, Daejeon, Korea) and sequenced bi-directionally using the M13F and M13R primers (Bioneer, Daejeon, Korea).

**Table 1:** Oligonucleotide primers used in cloning of cysteine protease.

Primer name	Sequence (5’-3’)
GSP-F	CATATCCATTGCCGATGAATCC
GSP-R	GTATTTAAAACACCATCGTACACAC
pET29b-propeptide-F	AAGGAGATATACATATGTACGAGGAATGGATAGTGAA
pET29b-protease-F	AAGGAGATATACATATGTTTCCCGTGCCTCCTTCC
pET29b-R	GGTGGTGGTGCTCGAGAAGGATGCTGATATGTCCT

### Construction of recombinant plasmids

New specific primers were designed from the sequence and used for subsequent PCR amplification of cDNA sequences with or without the pro-peptide region (Table 1). In-Fusion HD Cloning sites were introduced at the 5’ ends of pET29b-propeptide-F (forward primer), pET29b-protease-F (forward primer), and pET29b-R (reverse primer). The cDNA fragments were amplified by PCR and the pET29b(+) vector was linearized by double digestion using *Nde*I and *Xho*I (Takara Bio). After DNA purification, the PCR products were cloned with linearized pET29b(+) vector. Competent *Escherichia coli* DH5α cells were then transformed with the recombinant vectors, which were sequenced bi-directionally using T7 promoter and terminator primers.

### Molecular modeling

The three-dimensional structures of pro-SnuCalCp03 and pept-SnuCalCp03 were predicted by ModBasae search (http://modbase.compbio.ucsf.edu). Subsequently, all preliminary models have been evaluated in SAVES server (http://services.mbi.ucla.edu/SAVES). The models generated for pro-SnuCalCp03 and pept-SnuCalCp03 were prioritized on the basis of MPQS, discrete optimized protein energy (DOPE), and GA341 score. Then, the energy of final models was minimized by AMBER (http://ambermd.org). The final models were analyzed with PROCHECK and ERRAT plot, and graphic image was produced by PYMOL (http://www.pymol.org/). The root mean square deviation (RMSD) was determined simultaneously.

### Fusion protein expression

For fusion protein expression, *E*. *coli* BL21 (DE3) Star competent cells were transformed with confirmed pET29-propeptide and pET29-protease recombinant plasmids. A single colony was cultured overnight at 37°C in 5 mL of LB broth containing 50 μg/mL kanamycin. The overnight culture was subcultured using 1% inoculum to 100 mL of fresh LB broth containing the same concentration of kanamycin and grown at 37°C with continuous shaking at 220 rpm until the optical density at 600 nm reached 0.6–0.8. The transformed culture was induced with 0.5 mM isopropyl β-D-thiogalactopyranoside (IPTG) and incubated at 18°C. Cells were collected after 12 h incubation and the expression profile was assessed by SDS-PAGE and Western blot.

## Results and Discussion

### Sequencing and assembly

A total of 195,859,600 sequencing paired-end reads were generated. After trimming the adapter sequences and removing low-quality sequences, 171,253,393 clean reads remained for the normalized cDNA library, with an average GC content of 42.99% (Table 2). All sequence data have been deposited in NCBI Sequence Read Archive (SRA) under the study accession number SRP043253. Clean reads were assembled into 106,093 contigs longer than 200 bp using Trinity. These contigs had an average length of 1,614 bp, N50 of 2,703 bp, and maximum length was 16,561 bp. There were 57,177 contigs with lengths >1,000 bp and 33,827 contigs with lengths >2,000 bp (Table 2). In Velvet-Oases, considering N50, average contig length, max length, the number of contigs, and total length, we concluded that k-mer = 63 and 65 represented high connectivity of contigs and stable gene-sequence, respectively (S2 Table). We combined the contigs generated by Velvet-Oases using k-mer = 63 and 65, and assembled them again using Velvet followed by Oases to construct extended contigs. The 70,797 contigs assembled by Velvet-Oases were longer than 200 bp and had an average length of 1,565 bp, N50 of 2,082 bp, and maximum length was 13,520 bp. There were 44,490 contigs with lengths >1,000 bp and 18,658 contigs with lengths >2,000 bp (Table 2). Transcriptome Shotgun Assembly projects have been deposited at DDBJ/EMBL/GenBank under the accession GBHG00000000 (Trinity) and GBZK00000000 (Velvet-Oases). The versions described in this paper are the first versions, GBHG01000000 and GBZK01000000. To assess the final assembly, we re-aligned all reads with the contigs from the assembly using Bowtie2 (v2.0.0 beta5). The reads of 95.5% could be re-aligned with no more than one mismatch, which demonstrated that almost all reads were utilized for the *de novo* assembly.

**Table 2:** Summary of *Calotropis procera* transcriptome sequencing data and *de novo* assembly.

Items	Characteristics	
	Trinity	Velvet-Oases
Total number of reads	195,859,600	
Total number of clean reads	171,253,393	
GC percentage	42.99%	
Q20 percentage	96.62	
Total number of contigs	106,093	70,797
Average sequence size of contigs	1,614	1,565
N50 of contigs	2,703	2,082
Maximum sequence size of contigs	16,561	13,520
Contigs >1,000 bp	57,177	44,490
Contigs >2,000 bp	33,827	18,658

### Identification of cysteine protease genes and qRT-PCR validation

We obtained 15 and 20 different cysteine protease gene sequences from Trinity and Velvet-Oases, respectively (S3 Table). Although we could find more cysteine protease using Velvet-Oases than Trinity, the proteases lacked signal sequence and inhibitor region which were found in Trinity. Therefore, the cysteine proteases genes of the identical amino acid sequences were merged to construct extended transcripts containing full open reading frames. Few nucleotide differences without amino acid sequence changing were found and the full length cDNA sequences were selected. Consequently, we obtained 20 different cysteine proteases containing 17 full ORFs and three partial sequences (S4 Table). Although the assembled contigs with k-mer values from 45 to 75 were investigated in order to recover unique transcripts and partial sequences, we could not find new information. The cysteine proteases were named consecutively from SnuCalCp01 to SnuCalCp20 and their homology was analyzed against the GenBank database (by BLASTP) and InterProScan 4. The Characteristics of the cysteine proteases including protein length, BLASTP best match, and putative domains are shown in Table 3. All the cysteine proteases commonly contained peptidase C1A domain in their mature protein sequences and this result strongly suggested *Calotropis procera* cysteine proteases share common catalytic properties with papain family cysteine proteases. The 17 cysteine proteases contained I29 pro-peptide and signal peptide sequences, however, SnuCalCp20 contained no detectable pre-pro-domain and SnuCalCp06 and SnuCalCp10 contained only the peptidase C1A domain.

**Table 3:** Catalogue of cysteine protease encoding transcripts from *Calotropis procera*.

Unigene ID	Protein length (amino acids)	BLASTP best match (Species, Gene bank accession ID)	Identity	E-value	Putative domains contained
SnuCalCp01	354	Procerain B, partial (*Calotropis procera*, AGI59309.1)	208/212 (98%)	1e-151	Signal sequence, I29, peptidase C1A
SnuCalCp02	461	Cysteine protease Cp4 (*Actinidia deliciosa*, ABQ10202.1)	333/460 (72%)	0.0	Signal sequence, I29, peptidase C1A
SnuCalCp03	344	Cysteine protease CP15 (*Nicotiana tabacum*, AGV15823.1)	189/354 (53%)	7e-117	Signal sequence, I29, peptidase C1A
SnuCalCp04	373	Cysteine proteinase 15A-like (*Citrus sinensis*, XP_006473584.1)	281/344 (82%)	0.0	Signal sequence, I29, peptidase C1A
SnuCalCp05	458	Cysteine protease CP6 (*Nicotiana tabacum*, AGV15820.1)	337/443 (76%)	0.0	Signal sequence, I29, peptidase C1A
SnuCalCp06	297	Cathepsin L-like proteinase (*Medicago truncatula*, XP_003626102.1)	224/326 (69%)	7e-175	Peptidase C1A
SnuCalCp07	388	Papain-like cysteine proteinase (*Ipomoea batatas*, AAF61440.1)	295/372 (79%)	0.0	Signal sequence, I29, peptidase C1A
SnuCalCp08	363	Cysteine protease (*Nicotiana tabacum*, BAA96501.1)	286/363 (79%)	0.0	Signal sequence, I29, peptidase C1A
SnuCalCp09	362	Xylem cysteine proteinase 1-like (*Solanum tuberosum*, XP_006342169.1)	278/362 (77%)	0.0	Signal sequence, I29, peptidase C1A
SnuCalCp10	317	Pro-asclepain f (*Gomphocarpus fruticosus*, CAR31335.1)	253/316 (80%)	5e-176	I29, peptidase C1A
SnuCalCp11	367	Cysteine proteinase-like (*Vitis vinifera*, XP_002279940.1)	245/329 (74%)	0.0	Signal sequence, I29, peptidase C1A
SnuCalCp12	349	Cysteine protease CP15 (*Nicotiana tabacum*, AGV15823.1)	190/360 (53%)	5e-119	Signal sequence, I29, peptidase C1A
SnuCalCp13	382	Papain family cysteine protease (*Arabidopsis thaliana*, NP_567010.5)	246/351 (70%)	4e-180	Signal sequence, I29, peptidase C1A
SnuCalCp14	455	Cysteine protease family protein (*Populus trichocarpa*, XP_002307688.2)	280/417 (67%)	0.0	Signal sequence, I29, peptidase C1A
SnuCalCp15	366	Cysteine protease CP15 (*Nicotiana tabacum*, AGV15823.1)	186/359 (52%)	7e-118	Signal sequence, I29, peptidase C1A
SnuCalCp16	361	Cysteine protease (*Nicotiana tabacum*, BAA96501.1)	241/360 (67%)	0.0	Signal sequence, I29, peptidase C1A
SnuCalCp17	469	Cysteine protease Cp6 (*Actinidia deliciosa*, ABQ10204.1)	221/321 (69%)	3e-153	Signal sequence, I29, peptidase C1A
SnuCalCp18	356	Vignain-like (*Solanum lycopersicum*, XP_004239155.1)	280/353 (79%)	0.0	Signal sequence, I29, peptidase C1A
SnuCalCp19	363	Cys endopeptidase family protein (*Populus trichocarpa*, XP_002321654.1)	273/360 (76%)	0.0	Signal sequence, I29, peptidase C1A
SnuCalCp20	390	KDEL-tailed cysteine endopeptidase CEP1-like (*Solanum lycopersicum*, XP_004252607.1)	237/320 (74%)	6e-167	I29, peptidase C1A

**Fig 1:**
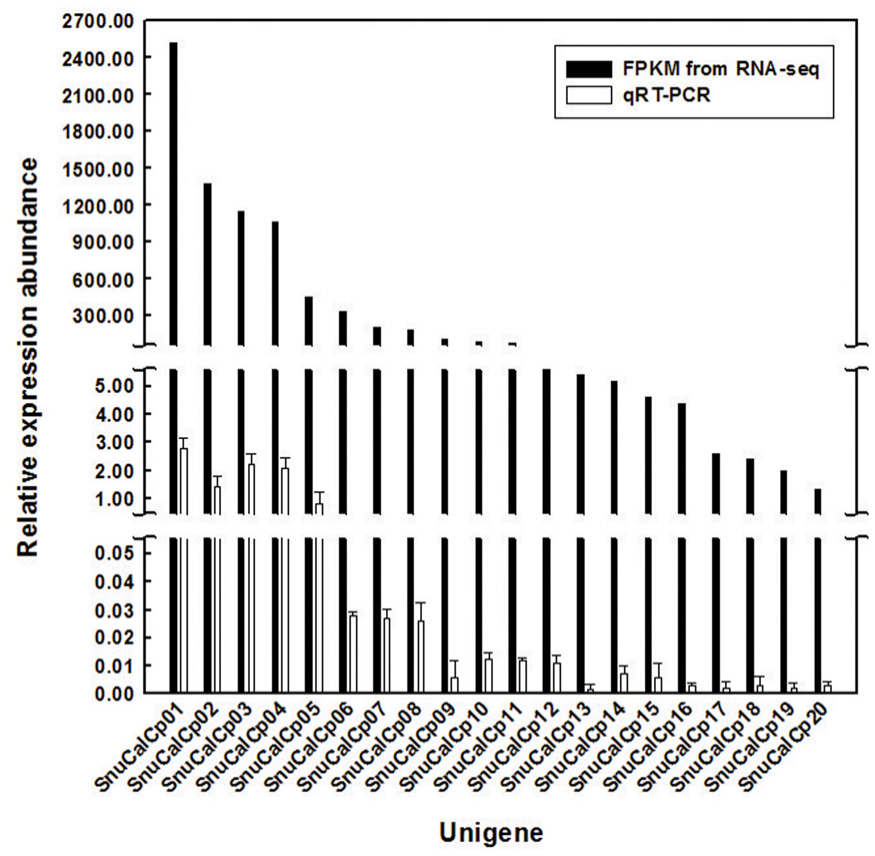
FPKM values of cysteine protease transcripts obtained from the *Calotropis procera* leaf and qRT-PCR validation.

SnuCalCp01 showed maximum sequence similarity (98%) with Procerain B, and an N-terminal sequence (FPVPCSVDWREKGALVPIKNQGRCGSCWAF) of CpCp-1, CpCp-2, and CpCp-3 was similar (99% identical) to a partial amino acid sequence of the SnuCalCp03. The abundances of the filtered data, expressed as fragments per kilobase of exon per million fragments mapped (FPKM), are shown in Fig. 1. The most highly expressed transcript, SnuCalCp01, encoding Procerain B, had an expression abundance of 2,535 FPKM, while the least highly expressed transcript, SnuCalCp20, encoding KDEL-tailed cysteine endoprotease CEP1, had an abundance of 1.1 FPKM. SnuCalCp03, which is considered a CpCp-3 due to its similar molecular weight, had the third highest expression level (1,093 FPKM). qRT-PCR data confirmed expression pattern of these unigenes by FPKM analysis. The results showed that the expression patterns of twenty unigenes were consistent with RNA-seq analysis.

### Multiple alignment and phylogenetic relationship

**Fig 2:**
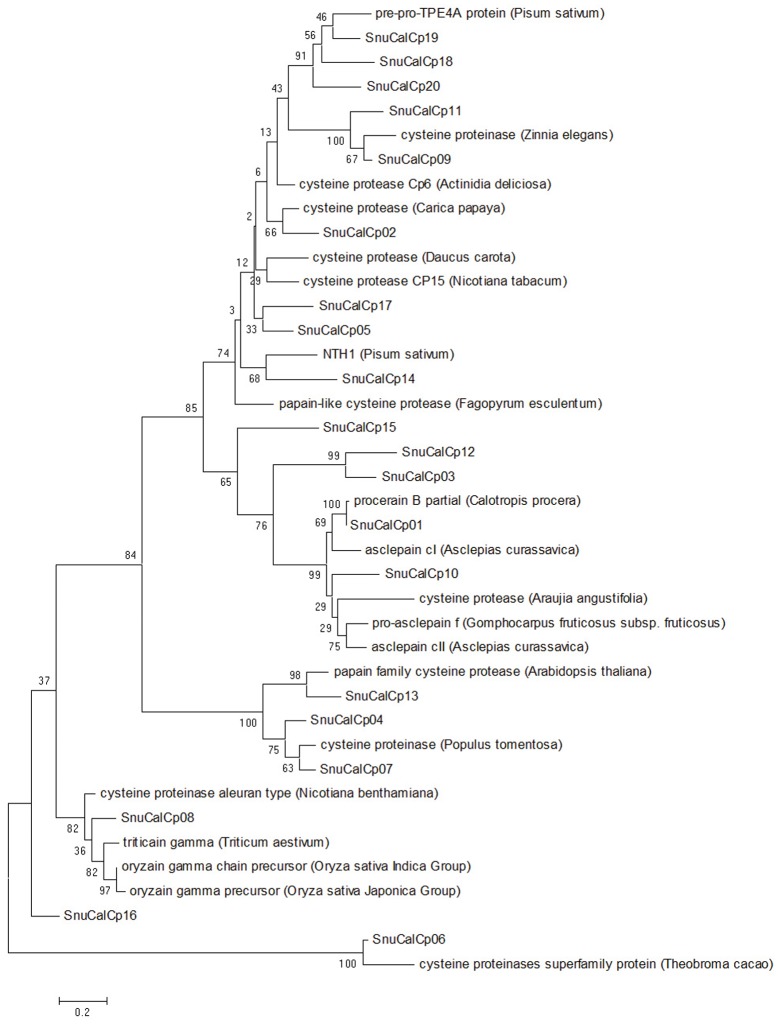
Phylogenetic analysis of SnuCalCps with other cysteine proteases. Evolutionary feature for multiple alignment of amino acid sequence was constructed with the NCBI database restricted to the Viridiplantae kingdom.

Identified cysteine protease sequences were classified into two main domains according to their general attribution: pro-region and protease. A phylogenetic tree inferred by Maximum Likelihood showed that the cysteine proteases (SnuCalCp01 and SnuCalCp03) form a separate group; moreover this cluster contained two distinct subgroups: SnuCalCp03 on one side and SnuCalCp01, pro-asclepain f (*A*. *fruticosa*), asclepain cll (*A*. *curassavica*), asclepain cl (*A*. *curassavica*), procerain B (*C*. *procera*), and cysteine protease (*A*. *angustifolia*) on the other (Fig. 2). It is noteworthy that proteases from latex (Asclepiadaceae and Caricaceae family) formed a group apart that would indicate a common ancestor and evolution with inhibitors. An amino acid group sequence alignment of cysteine proteases with papain (GenBank: AAB02650) from *Carica papaya* showed that the consensus sequence of cysteine proteases belongs to subfamily C1A (papain family) (S1 Fig.) [21,22].

These proteases contain the ERFNIN (**E**XXX**R**XXX**F**XX**N**XXX**I**XXX**N**) motif, which is a highly conserved motif, this motif is present in the long α-helix which contributes a major part in the scaffold of the cathepsin L like pro-peptide. Another highly conserved GNFD (**G**X**N**X**F**X**D**) motif is also found for this family. This motif is located at the kink of the β-sheet ahead of the short α-helix (α3) capping the opening of the interdomain cleft. The detection of ERFNIN-GNFD motif in the pro-region of cysteine proteases clearly implies its relationship to the cathepsin L group and sets it apart from cathepsin B subfamily [23–25].

Like all papain superfamily members, the proteins encoded by cysteine proteases harbor the conserved residues associated with catalysis (also known as the catalytic triad), namely Cys25, His159, and Asn175 (papain numbering) as well as the six cysteine residues known to be involved in the formation of the disulfide bond. The Cys25 is embedded within a highly conserved peptide sequence, CGSCWAFS. The His159 is adjacent to small amino acid residues, such as glycine or alanine, and Asn175 is a portion of the Asn-Ser-Trp motif.

### Cloning and molecular modeling of SnuCalCp03

**Fig 3:**
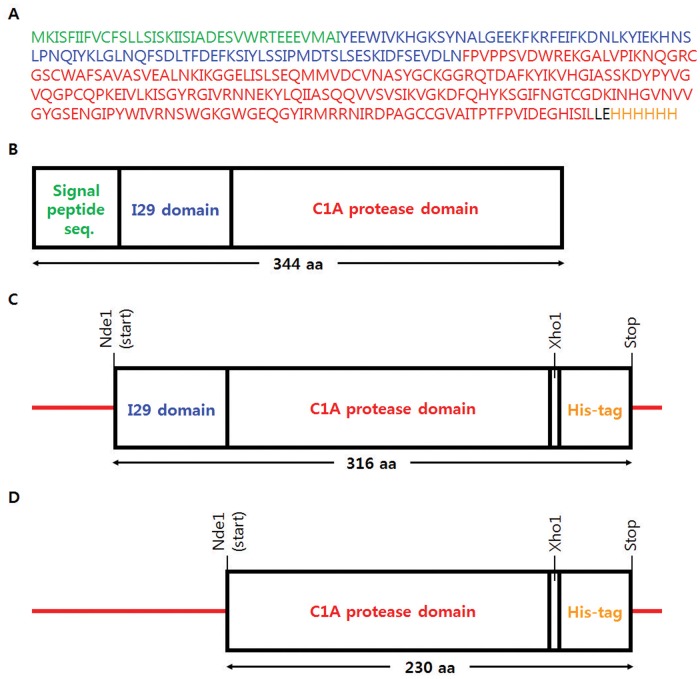
Amino acid sequence and recombinant protein construction of SnuCalCp03. (A) Deduced amino acid sequence of open reading frame for SnuCalCp03 and putative domains of (B) pre-pro-SnuCalCp03, (C) recombinant pro-protease with 6X histidine tag, and (D) recombinant protease with 6X histidine tag.

In order to express and characterize the cysteine proteases of *Calotropis procera*, we selected SnuCalCp03 that had been previously investigated as a purified mature protease. Fig. 3A and B shows SnuCalCp03 consists of pre- (28 amino acids), pro- (86 amino acids), and mature (218 amino acids) parts. Both the proteins, recombinant SnuCalCp03 with I29 pro-peptide (pro-SnuCalCp03, Fig. 3C) and without I29 pro-peptide (pept-SnuCalCp03, Fig. 3D), were constructed with deduced amino acid sequence.

**Fig 4:**
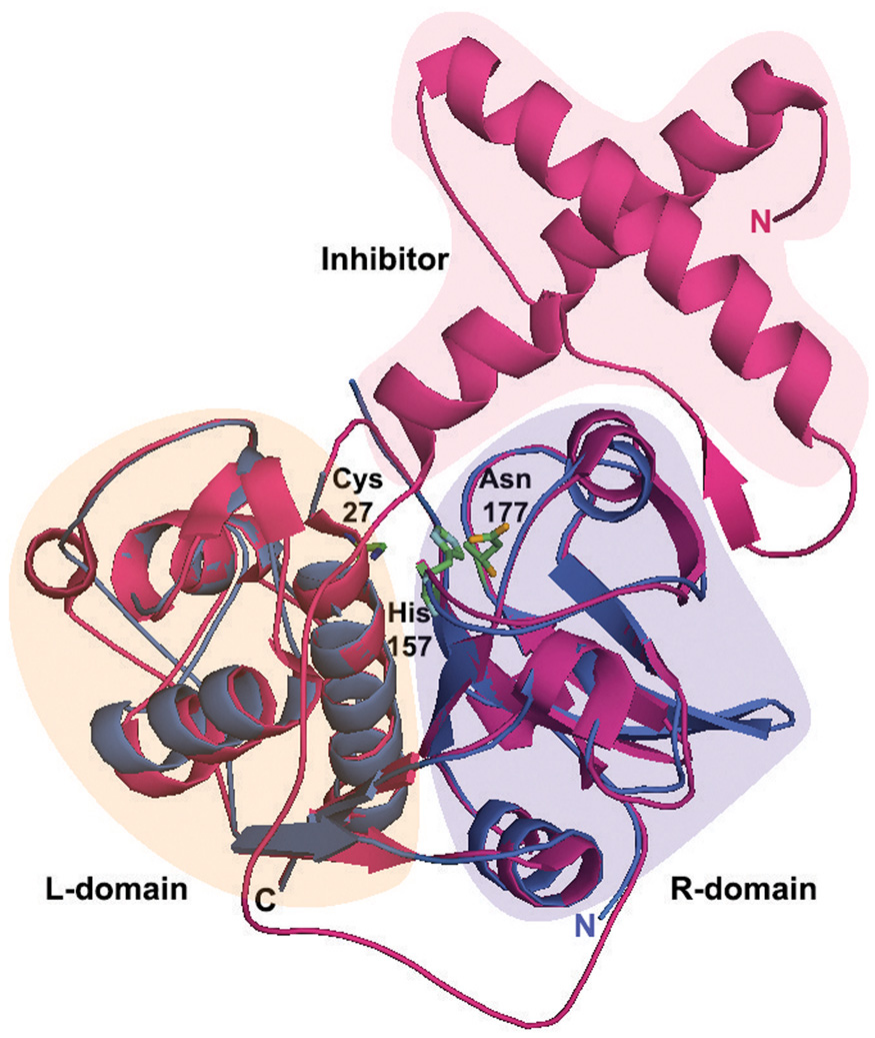
The three dimensional structure modeling of pre-mature (red) and mature (blue) SnuCalCp03. The three dimensional structures were modeled with Modeller software using the X-ray crystal structure of pro-papain (red; PDB ID: 3TNX) from Carica papaya and GP-II (blue; PDB ID: 1CQD) from Zingiber officinale. Structure-based sequences were aligned with PyMol software and the model revealed interactions between the inhibitor and active site cleft (Cys27, His157, Asn177).

Structural analysis of pro-SnuCalCp03 and pept-SnuCalCp03 was performed, which helps us to understand the zymogen structure and the activation mechanism of SnuCalCp03 itself (Fig. 4). Sequence analysis of pro-SnuCalCp03 showed 46% identity with pro-papain (PDB ID:3TNX) from *Carica papaya* whereas the identity of pept-SnuCalCp03 was 53% with GPII (PDB ID:1CQD) from *Zingiber officinale*. Thus, two aforementioned structures were selected as templates to construct the three-dimensional structure of pro-SnuCalCp03 and pept-SnuCalCp03, respectively. The quality of final model was validated by Ramachandran plot analysis with PROCHECK and ERRAT plot. PROCHECK analysis showed that residues of pro-SnuCalCp03 and pept-SnuCalCp03 in the most favorable region were 82.8% and 86.2%, and in the additional allowed region were 14.2% and 13.7%, respectively. The overall quality factor of the model from ERRAT plot for pro-SnuCalCp03 and pept-SnuCalCp03 were 93.7 and 86.4, respectively, indicating that the modeled structure had low steric hindrance (most of the residues were below 95% cut-off of error-value). Furthermore, structural comparison between pro-SnuCalCp03 and pept-SnuCalCp03 showed that both the proteins were superimposed completely with RMSD of 1.04 A° over 208 residues. The mature part is subdivided into two typical domains referred to as the left domain (L-domain) and the right domain (R-domain) of papain-like cysteine proteases sharing “V” shaped active site comprised of Cys27, His157, and Asn177. Modeled structure of pept-SnuCalCp03 is structurally similar to the mature part of pro-SnuCalCp03 and both the proteins are almost superimposable, revealing that inhibitor poses steric hindrance to substrate binding at the catalytic cleft. The structural analysis demonstrated that the pro-peptide of the zymogen undergoes a rearrangement in the form of a structural loosening at acidic pH which triggers the proteolytic activation cascade [26,27]. We therefore attempted to express pro-SnuCalCp03 and pept-SnuCalCp03, and to induce autocatalytic activation at the acidic pH.

### Expression, purification, and refolding of SnuCalCp03

**Fig 5:**
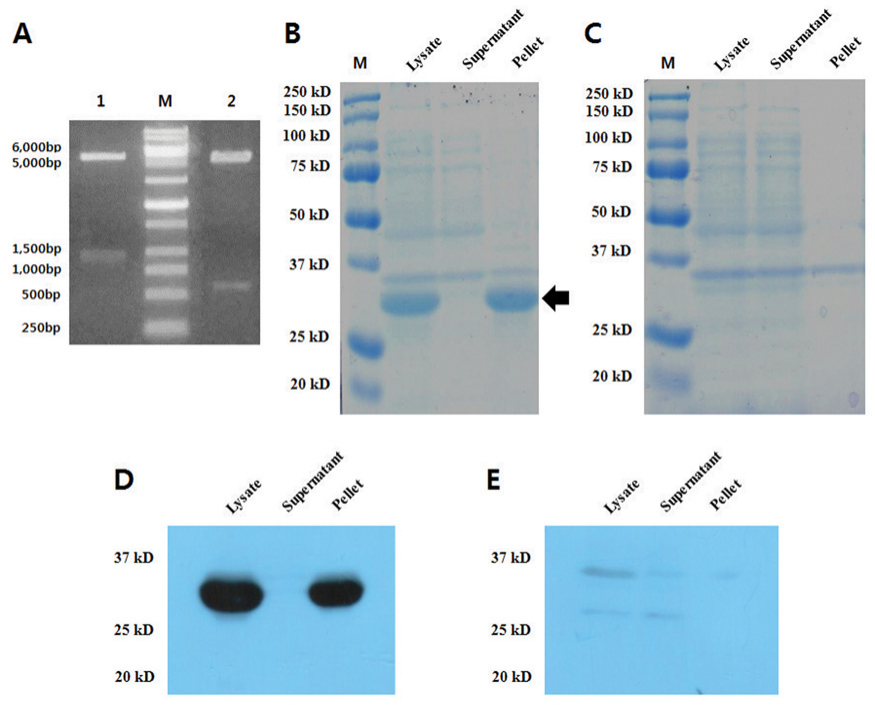
Confirmation of pET29b-zymogen and pET29b-protease clones with double digestion and expression test. (A) Lane 1 represents release of 1,056 bp fragment (zymogen cDNA) and Lane 2 represents release of 663 bp (protease cDNA) fragment on double digestion (NdeI/XhoI) of pET29b-construct. Expression of recombinant (B) zymogen and (C) protease in BL21 was analyzed on SDS-PAGE. Expressed recombinant (D) zymogen and (E) protease were validated by Western blot analysis.

In order to confirm the expression level of recombinant proteins encoding partial sequences of SnuCalCp03 in *E*. *coli* with and without a propetide sequence, we obtained a 1,079 bp cDNA and a 660 bp cDNA fragment containing a zymogen domain and protease C1A domain, respectively (Fig. 5A). The cDNA fragment was cloned into a T-blunt vector to form a recombinant structure and then sequenced. There were no mismatches compared with RNA-Seq data. To express the recombinant proteins, the cDNA fragment was amplified with gene-specific forward and reverse primers containing appropriate In-Fusion cloning sites and subcloned into the pET29b(+) expression vector. The recombinant zymogen and protease were expressed in *E*. *coli* BL21 (DE3) Star and the cultures were sonicated to release the recombinant proteins. SDS-PAGE (Fig. 5B and Fig. 5C) and Western blot (Fig. 5D and Fig. 5E) analysis of the lysate revealed that the expressed zymogen had an approximate molecular weight of 35 kDa; no protease was detected. Although it is possible to express to the enzyme without its pro-peptide domain, yields are extremely low compared with expression of the zymogen. This result indicates that expression of cysteine proteases may be strongly related to the presence of the pro-peptide which prevents cleavage of structural cell wall proteins such as extensins, thus contributing to the final cell collapse [28]. Therefore, successful protocols use zymogen cDNAs encoding pro-peptide, which are cloned into an expression vector. Three *E*. *coli* strains (Origami, C41, C43) with and without pelB leader sequence to induce proper folding were also tested for protease expression, but the protein was not expressed without pro-peptide domain. Most of the recombinant zymogen was expressed in form of inclusion bodies present in pellet and those were dissolved with 8 M urea and purified by Ni-affinity chromatography. Attempts to oxidative refolding of enzyme were made using reduced and oxidized glutathione. Autocatalytic activation of zymogen was processed at pH 4.0, but enzyme was still found in non-active form. The lack of proteolytic activity may be attributed to misfolding of protein due to the absence of proper folding conditions during *in vitro* refolding.

### Conclusions

The genetic information and the expression pattern of recombinant protease obtained from this study could help to design biologically active cysteine proteases with inhibitors, which is essential for the correct folding, transport, maturation, and regulation of activity, although further studies to elucidate the exact roles of domains for these proteases should be conducted.

## Supporting Information

S1 Fig Multiple alignment analysis of deduced amino acid sequences of SnuCalCps with Papain. The deduced amino acid sequences of (A) pro-peptide domain and (B) mature domain were aligned by ClustalW. Identical and conserved amino acid residues are darkly shaded, and the amino acids numbers are shown on the left. Conserved signatures (ERFNIN and GNFD) and catalytic triad residues (C, H, and N) are highlighted in bold and indicated above the alignment.
(PDF)

S1 Table List of primer sequences designed for qRT-PCR. (DOCX)

S2 Table Summary statistics of the assemblies of the Calotropis procera sequence data showing the performances of different k-mer length. (DOCX)

S3 Table Putative domain of cysteine proteases from de novo assemblies. (DOCX)

S4 Table Nucleotide sequences of cysteine proteases. (DOCX)
