# Supplementary material for: Cysteine Protease Profiles of the Medicinal Plant Calotropis procera R. Br. Revealed by De Novo Transcriptome Analysis
Source: PLoS One. 2015 Mar 18;10(3):e0119328. doi: 10.1371/journal.pone.0119328 (PMC4365007; doi:10.1371/journal.pone.0119328)
Supplement: S1 Fig — The deduced amino acid sequences of (A) pro-peptide domain and (B) mature domain were aligned by ClustalW. Identical and conserved amino acid residues are darkly shaded, and the amino acids numbers are shown on the left. Conserved signatures (ERFNIN and GNFD) and catalytic triad residues (C, H, and N) are highlighted in bold and indicated above the alignment. (PDF) [file pone.0119328.s001.pdf]

```
A                                E    R   F  N   I   N            G N F D
     Papain (1) FESWMLKHNKIYKNIDE-KIYRFEIFKDNLKYIDETNK----KNNSYWLGLNVFADMSNDEF
SnuCalCp09  (1) FESWIEKHGKIYETIEE-KLHRFEIFKENLKHIDERNK----IISNYWLGLNEFADLSHEEF
SnuCalCp11  (1) FESWASKHGKRYKSVEE-KLLRFENFMDNLKHIDESNKE---ANKTYWLGLNEFADLSHEEF
SnuCalCp04  (1) FSLFKAKFGKSYASLEE-HDYRLSVFKANLRRAKRNQILDP----SAVHGVTKFSDLTPSEF
SnuCalCp07  (1) FSIFKKKFGKSYVSIEE-ENYRLSVFKANMRRAQRNQE----LDPSAEHGVTQFSDLTPREF
SnuCalCp13  (1) FKSFVEEYGKEYSSRKE-YVHRLGVFAKNLLRAAEHQAMDP----TAVHGVTEFSDLTEDEF
SnuCalCp08  (1) FARFAHRYGKRYETAEE-IKVRFDIFRDNLRMIKSHNKK----GLSFSLGVNAFSDLTWEEF
SnuCalCp16  (1) FASFALKYGKKYETDGE-IKKRFEIFRENLWKIESHNKK----GLSYTLGINEFSDMTWEEF
SnuCalCp14  (1) FENWCREYGKTYSSEQE-KQYRHGVFKDNYDYITQHNSK---GNSTCTLSLNAFADLTHHEF
SnuCalCp18  (1) YEKWRSHHTVSRDLTEK--QKRYNVFKANALHVYNSNK----MDKPYKLKLNKFADMTSHEF
SnuCalCp19  (1) YERWRSHHTISTNLDEK--HRRFNVFRANAHYVHEFNKK---DDVPYKLKLNKFADMTNHEF
SnuCalCp20  (1) HEQWMVQYGRVYKDDTE-KASRFKIFKENVEYIESFNKAG---IRSYKLGINKFADLTNEEF
SnuCalCp01  (1) YEEWLVKHQKLHSSLGE-KIKRFEIFKDNLRYIDEQNNYNKFNHKNFTLGLNQFADLTLDEF
SnuCalCp10  (1) YEEWLVKHQKLHSNLGE-KIKRFKIFKDNLRYIDEQNNYNKVNHMNFTLGLNQFADLTLDEF
SnuCalCp02  (1) YESWLVKHGKAYNALGE-KEKRFEIFKDNLQFIDEHNS----KNLSYKLGLNRFSDLSHEEF
SnuCalCp05  (1) YEAWLVEHGKSYNGLGE-KDKRFEIFKDNLRYIDEQNSV---ANRTYKLGLNRFADLTNDEY
SnuCalCp17  (1) YEEWLVKHGKAYNALGE-KSNRFAIFKDNLRYIDDHNSN---ANRTYKLGLNQFADLTNSEF
SnuCalCp03  (1) YEEWIVKHGKSYNALGEEKFKRFEIFKDNLKYIEKHNSL---PNQIYKLGLNQFSDLTFDEF
SnuCalCp12  (1) YEEWMVEYRKSYDALGVEKLKRFEIFKDNLKYMEEHNSL---PNQTYKLGLNQFSDLTLREF
SnuCalCp15  (1) FEEWLVKHRKVYNAIGE-KEKRFEIFKNNLKFIDEHNIR--YPNKTYTLGLNVFADLTDDEY
```

B

```
                                    25
Papain      (1) ----IPEYVDWRQKGAVTPVKNQGSCGSCWAFSAVVTIEGIIKIRTGNLNEYSEQELLDCDRR---------SYGCNGGYPWSALQLVAQYG-IHYRNTYPYEGVQRYC
SnuCalCp04  (1) ----LPTDFDWRDHGAVTGVKDQGSCGSCWSFSATGALEGAHYLATGELVSLSEQQLVDCDHECDPEESGSCDSGCNGGLMNNAFEYVLKAGGVVSEQTYPYTGTDGKC
SnuCalCp07  (1) ----LPSDFDWRDRGAVGPVKNQGSCGSCWSFSTTGALEGANYLATGKFVSLSEQQLVDCDHECDPAEADSCDSGCNGGLMNNAFQYTLKAGGIMREEDYPYTGTDRGT
SnuCalCp13  (1) ----LPENFDWRDHGAVTEVKTQGTCGSCWAFSTTGAIEGANFVATGKLVSLSEQQLVDCDHACDIKDKDSCDDGCSGGLMTNAYNYLIEAGGIEEESSYPYTGKRGEC
SnuCalCp06  (1) LPNQFDARTAWPQCATIGRILDQGHCGSCWAFGAVETLSDRFCIHFGMNISLSVNDLLSCCGFMCG-------NGCDGGYPLSAWKYFVYSG-VVTEECDPYFDNEGCS
SnuCalCp08  (1) ----LPEMKDWRETGIVSPVKDQGHCGSCWTFSTTGALEAAYTQAFKKEISLSEQQLVDCAGAFN-------NFGCRGGLPSQAFEYIKYNGGLDTEEAYPYVGKNGVC
SnuCalCp16  (1) ----LPKSKDWRDAGIVSPVKDQGNCGSCWTFSTTGALEAAYAQAYGQNISLSEQQLLDCAGDFN-------NFGCDGGLPSQAFEYIKYSGGIETEESYPYMEKEGEC
SnuCalCp01  (1) ----LPNSVDWRKKDVVFPIRNQGQCGSCWTFSAVASIETLIGIKEDRMIALSEQELLDCERTS---------YGCKGGYYTNAFAYVAKKG-LTSREKYPYIFQQGQC
SnuCalCp10  (1) ----LPKSVDWRKKGFVLPIRNQKKCGACWAFSAVASIETLIGIKKGRTIALSEQELLDCVTAC---------DGCKAGHYDSAFAYVAKYG-ITSREKYPYVYKKGQC
SnuCalCp03  (1) --FPVPPSVDWREKGALVPIKNQGRCGSCWAFSAVASVEALNKIKGGELISLSEQMMVDCVNAS---------YGCKGGRQTDAFKYIKVHG-IASSKDYPYVGVQGPC
SnuCalCp12  (1) ----LPDSVDWRKRGAVLPIKNQGSCGSCWAFSAIASVEALNKIKGGDLISLSEQMLVDCVTS---------SHGCNGGRQIDAFRYMTDHG-IASSDDYPYEAVRGSC
SnuCalCp15  (1) ----LPDFVDWRSKGIVLPIENQGDCGSCWAFSAICSVEGIYAQRSGKLISLSKQELVDCERR---------SYGCDGGDYSTAFEYITFFG-VSSEKSYPYTAEDGDC
SnuCalCp02  (1) ----LPKSVDWREKGAVAPVKDQGQCGSCWAFSTVGAVEGINKIVTGDLVTLSEQELVDCDNTY--------NQGCNGGLMDYAFEFITNNGGIDTEDDYPYTARDGTC
SnuCalCp05  (1) ----LPDSVDWRKEGAVVPVKDQGSCGSCWAFSTIASVEGISAVVTGDLISLSEQELVDCDISY--------NEGCNGGLMDYAFQFIIDNGGIDSEEDYPYTAKDGRC
SnuCalCp17  (1) ----LPDSVDWREKGAVVPIKDQGSCGSCWAFSAISAVEGINAIVTGTLISLSEQELVDCDNSGG-------NEGCSGGLMDPAFEFITENGGIDSEEDYPYKGRQGTC
SnuCalCp09  (1) ----IPKSVDWRKKGAVTPVKNQGSCGSCWAFSTVAAVEGINQIVTGNLTSLSEQELIDCDTAY--------NNGCNGGLMDYAFSFIVSNGGLHKEEDYPYLMEEGTC
SnuCalCp11  (1) ----IPKSMDWRKRGAVGHVKNQGPCGSCWAFSTVAAVEGINKIVTGNFTSLSEQELIDCDTS-F-------NNGCNGGLMDYAFQYITQNGGLRKEEDYPYLMDQGTC
SnuCalCp14  (1) -------------------------------------MEGINKIVTGSLISLSEQELIDCDRSY--------NSGCEGGLMDYAYKFVVNNNGIDTENDYPFQGRSGAC
SnuCalCp18  (1) ----LPTSVDWRKQGAVTGVKDQGRCGSCWAFSTVVGVEGINKIKTGHLISLSEQELVDCESD---------NEGCNGGLMENAYEFIKKKGGITTERAYPYRARNELC
SnuCalCp19  (1) ----VPTSIDWRQKGAVNPVKDQGQCGSCWAFSTVVAVEGINYIKTTKLVSLSEQELVDCDNRE--------NEGCNGGLMDVAFEFIMKNGGITTEQNYPYRARDGRC
SnuCalCp20  (1) ----VPASMDWRKKGAVTGIKDQGQCGCCWAFSAVAAMEGINQLTTHKLISLSEQELVDCDTSE--------DQGCNGGLMDDAFQFIISNKGLTTESNYPYQGVDGTC

                                                                                                      159
Papain     (96) RSREK-------------------GPYAAKTDGVRQVQPYNEGALLYSIANQPVSVVLEAAGKDFQLYRGGI-FVGPCGNK----VDHAVAAVGY-----------GPN
SnuCalCp04 (106) SFDK--------------------SKVVAKVANFSVVSLDEDQIAANLVKHGPLAVGINAA--WMQTYIGGVSCPYICGKH----LDHGVLLVGYGAEGYAPIRLKEKP
SnuCalCp07 (106) CKFDQ-------------------TKVAAKVANFSVVSLDEDQIAANLVKNGPLAVAINAVY--MQTYIKGVSCPYICSKR----LDHGVLLVGYGSEGYAPIRMKDKP
SnuCalCp13 (106) KFTP--------------------EKVAVRLQNFTTVSGSEEQIAAHLVRNGPLAVGLNAVF--MQTYIGGVSCPLICGKK---FLNHGVLLVGYGSSGFSIIRLGHKP
SnuCalCp06 (102) HPGCEPGYPTPKCHRKCVKGNLLWKQAKHYSKRPHKIKSDPYDIMAEVYKNGPVQVSFTVY-EDFAHYRSGVYKHITGSSLG----GHSVKLIGWGTS------EDGED
SnuCalCp08  (99) KYSSE-------------------NVGVRVLDSVNITLGAEDELKYAVGLLRPVSVAFEVVK-DFRHYKSGVYTSNTCGSSPMD-VNHAVLAVGYG-------VEDGIP
SnuCalCp16  (99) KFYSG-------------------NVAVRVRDSFNISQGNEYDLQVAVAYIRPVSVAFQVL-EDFKQYKSGIYTSTECGSTP-EDVNHAVLAVGFG-------IENGTP
SnuCalCp01  (96) YQ----------------------KEKVVKISGYRRIPKNDEKKLQSVVAQQVVSVGVKSKSRDFQHYRSGV-FSGACGPR----VDHAVNIVGYGS-------EGGVN
SnuCalCp10  (96) SR----------------------KKKVVKISNYKRLLRNNEGQLQIAVAQQVVSVGVKATSKDFQHYRSGI-FKGACGPQ----LNHAVNIVGYGS-------EGGVN
SnuCalCp03  (98) QP----------------------KEIVLKISGYRGIVRNNEKYLQIIASQQVVSVSIKVG-KDFQHYKSGI-FNGTCGDK----INHGVNVVGYGS-------ENGIP
SnuCalCp12  (96) KN----------------------KKIVVKIKGYRRILPRRERFLLSRASMQVVSVSIKAGSKDFQHYKSGI-FNGKCGNK----INHGVNVVGYGS-------EDGNA
SnuCalCp15  (96) KS--D-------------------MIKVVQIDGYYDINYNDEKALQKSVSEQVVSVAVKANSVEFKLYNSGI-FSGKCGIE----SDHAVNIIGYGS-------EDNVD
SnuCalCp02  (98) DQFRK-------------------NARAVSIDGFEDVPKGDEQSLKKAVAHQPVSIAIEAGGRAFQHYQSGV-FTGHCGTN----LDHGVVAVGYG-------TEDGVD
SnuCalCp05  (98) DQYRK-------------------NAKVVTIDGYEDVPRNNEEALKTAAASQVVSVAIEGGGRDFQLYRGGV-FTGKCGTS----LDHGVNVVGYGS-------ENGLD
SnuCalCp17  (99) DQYRV-------------------NVKVVKINGYEDVPRNNEKALKKAVASQVVSAAISAGGIDFQLYESGI-YSGRYGTE----LDHGISIVGYGS-------ENGYD
SnuCalCp09  (98) DEKRT-------------------ESEVVTISGYHDVPQSNEQSFLKALAHQPLSVAIEASGRDFQFYSGGV-FDGHCGTE----LDHGVAAVGYGS-------TKGLD
SnuCalCp11  (98) EATKE-------------------ETDVVTISGYQDVPQNDEQSLLKALAHQPLSVAIEASGRDFQFYKGGI-FTGPCGSQ----LDHGVAAVGYGS-------SKGLD
SnuCalCp14  (65) NRNKL-------------------KRRVVTIDGYRDVPAGNENALMKAVAVQPVSVGICGSERAFQLYSGGV-FTGPCSTS----LDHAVLIVGYDS-------HNGVD
SnuCalCp18  (97) DSEKM-------------------NAPVVKIDGHEMVPEDDEDALMKAVANQPVSVAIDASGINMQFYSEGV-FTGPCGTE----LDHGVAAVGYGT------TLDGTK
SnuCalCp19  (98) DKNKA-------------------SHSAVSIDGYEDVPANNEDALLKAVANQPVSVAIDAGGSDFQFYSEGV-FTGKCGTE----LDHGVAIVGYGT------TVDGTK
SnuCalCp20  (98) NSNKE-------------------SNSAAKITGYEDVPENSEAALLKAVANQPVSVAIDASGSDFQFYSSGV-FTGECGTD----LDHGVTAVGYGKS-----AEDGTK
```

```
                              175
    Papain  (170) YILIKNSWGTGWGENGYIRIKRGTGNSYGVCGLYTSSFYPV
SnuCalCp04  (189) YWIIKNSWGENWGEEGYYKICRG----RNVCGVDSMVSTVA
SnuCalCp07  (190) YWIIKNSWGEHWGENGFYKICRG----RNVCGVDSMVSTVA
SnuCalCp13  (190) YWIMKNSWGKRWGEHGYYRMCRG----HNMCGINTMVSAVV
SnuCalCp06  (200) YWLLANQWNKSWGDEGYFMIKRG----TNECGIEDSVVSGM
SnuCalCp08  (180) YWLIKNSWGASWGDNGYFKMEMG----KNMCGVSTCASFPV
SnuCalCp16  (180) YWIIKNSWGTDFGIHGYFMMEMG----KNMCGVATCASFPF
SnuCalCp01  (171) YWIVRNSWGTNWGENGYMRIPRNSNQSGGYCGIAVQAAYPV
SnuCalCp10  (171) YWIVRNSWGTGWGEKGYMRILRNSKQSEGYCGIAMKPSFPV
SnuCalCp03  (172) YWIVRNSWGKGWGEQGYIRMRRNIRDPAGCCGVAITPTFPV
SnuCalCp12  (171) YWIIRNSWGEKWGEQGYMRMPRNIRKSEGYCGIALRPSLPV
SnuCalCp15  (172) YWIVRNSWGTDWGENGYMRIIRNTKDPQGHCGIADEPSYPV
SnuCalCp02  (176) YWLVRNSWGPKWGEAGYIKLERNVHGNTGKCGIAMEPSYPV
SnuCalCp05  (176) YWIVRNSWGPSWGENGYIRMQRNVASSSGLCGIAIEPSYPI
SnuCalCp17  (177) YWILRNSWGTKWGEDGYMRLRRNVPESGGHCQIATLPSYPI
SnuCalCp09  (176) YIIVKNSWGPKWGEKGYIRMKRNTGKPEGICGINKMASYPT
SnuCalCp11  (176) YIIVRNSWGSTWGERGYIRMQRNTGNHEGLCGINKMASYPV
SnuCalCp14  (143) YWIVKNSWGTSWGIEGYAYMARNTGNSEGVCGINMLASYPV
SnuCalCp18  (176) YWIVKNSWGTEWGEGGYIRMVRGSHAEGGICGIAKEASYPV
SnuCalCp19  (177) YWIVRNSWGPEWGEKGYIRMHRGINAKEGLCGIAMQPSYPI
SnuCalCp20  (178) YWLVKNSWGTSWGEN--------------------------
```
