# Supplementary material for: Cysteine Protease Profiles of the Medicinal Plant Calotropis procera R. Br. Revealed by De Novo Transcriptome Analysis
Source: PLoS One. 2015 Mar 18;10(3):e0119328. doi: 10.1371/journal.pone.0119328 (PMC4365007; doi:10.1371/journal.pone.0119328)
Supplement: S1 Table — (DOCX) [file pone.0119328.s002.docx]

**S1 Table. List of primer sequences designed for qRT-PCR.**

|  |  |  |
| --- | --- | --- |
| **Unigene** | **Forward primer (5’ to 3’)** | **Reverse primer (5’ to 3’)** |
| GAPDH | GATCGGACAATTGGGATTTG | TTGCCCTGAGCAAGAACTTT |
| SnuCalCp01 | CACCCATAACTTGTTCTTTCGC | TCAAGGACAATGTGGGAGTTG |
| SnuCalCp02 | AGGGAGAAAGGTGCTGTTG | ACCTACTGTTGAGAATGCCC |
| SnuCalCp03 | ACGGATCTGAAAACGGGATT | ATAGCAACTCCGCAACAACC |
| SnuCalCp04 | GGATATGCACCTATTCGCCTC | AGCCACAGTTGAGACCATG |
| SnuCalCp05 | TGCCATTGAGCCTTCTTACC | ACAGCAACCCCATTCATAGC |
| SnuCalCp06 | GCCACAGCAAGATAGGAGATC | GGGAGAATTCTTGATCAGGGAC |
| SnuCalCp07 | CATTGCATCCCGAATCACATG | ACAACTGGAGCTTTGGAAGG |
| SnuCalCp08 | GTCTCGTACCTCTTTCCATACC | TGAATCCGATCAGGCAAGTC |
| SnuCalCp09 | GCTTTGTTCATTGCTCTGGGG | TCCAATGGTGGCCTTCACAA |
| SnuCalCp10 | AGGAAAAGAGGGCTTCATGG | GGATATGGTTCTGAAGGTGGAG |
| SnuCalCp11 | GCACTATCCCTTACAGATTCCC | CATCACTTTCAGCCCGTTTC |
| SnuCalCp12 | TCTTTGCCTGACTCCGTTG | AGCAACTCCCACAACTTCC |
| SnuCalCp13 | AAGCTGCTGCTCACTTAGGC | GCAGGGAACTTGTGGATCAT |
| SnuCalCp14 | GGTATTGCCAGTTCTCAC GAG | CTTGGGTTTTGCCTT TCATGG |
| SnuCalCp15 | GTTCAGTGGAAGGGATATACGC | ATAATCACCTCCATCGCATCC |
| SnuCalCp16 | CCGACGACTTCA AGGAAA GA | GTTTCTCTGATCGCCGTCTC |
| SnuCalCp17 | TCTCCCAAGGCATTATACGC | CTTCTGAGATCACGTCGGTTAG |
| SnuCalCp18 | GGTGGGATACGGAACAACAC | AATGCCTCCTTCAGCATGAC |
| SnuCalCp19 | AGAGCTCGCGATGGAAGATG | CATCAATGGCGACGGAAACG |
| SnuCalCp20 | CCCTGAAAATAGCGAAGCTG | CACCATGGTCCAAGTCTGTG |
