# Supplementary material for: Cysteine Protease Profiles of the Medicinal Plant Calotropis procera R. Br. Revealed by De Novo Transcriptome Analysis
Source: PLoS One. 2015 Mar 18;10(3):e0119328. doi: 10.1371/journal.pone.0119328 (PMC4365007; doi:10.1371/journal.pone.0119328)
Supplement: S2 Table — (DOCX) [file pone.0119328.s003.docx]

**S2 Table. Summary statistics of the assemblies of the *Calotropis procera* sequence data showing the performances of different k-mer length.**

|  |  |  |  |  |  |
| --- | --- | --- | --- | --- | --- |
| **k-mer** | **Contigs ≥ 200** | **N50** | **Average length** | **Max length** | **Total length** |
| 45 | 81,909 | 2,395 | 1,601 | 16,320 | 131,146,621 |
| 55 | 75,676 | 2,359 | 1,563 | 16,320 | 118,303,156 |
| 61 | 70,240 | 2,310 | 1,534 | 16,531 | 107,767,518 |
| 63 | 68,271 | 2,268 | 1,514 | 16,468 | 103,385,465 |
| 65 | 65,828 | 2,226 | 1,499 | 16,466 | 98,688,437 |
| 67 | 63,785 | 2,193 | 1,474 | 16,430 | 94,066,067 |
| 69 | 61,438 | 2,174 | 1,457 | 16,431 | 89,522,905 |
| 75 | 53,634 | 2,069 | 1,395 | 13,573 | 74,870,258 |
