# Supplementary material for: Cysteine Protease Profiles of the Medicinal Plant Calotropis procera R. Br. Revealed by De Novo Transcriptome Analysis
Source: PLoS One. 2015 Mar 18;10(3):e0119328. doi: 10.1371/journal.pone.0119328 (PMC4365007; doi:10.1371/journal.pone.0119328)
Supplement: S3 Table — (DOCX) [file pone.0119328.s004.docx]

**S3 Table. Putative domain of cysteine proteases from *de novo* assemblies.**

|  |  |  |
| --- | --- | --- |
| Unigene ID | Putative domains contained | |
|  | Trinity | Velvet-Oases |
| SnuCalCp01 | Signal sequence, I29, peptidase C1A | Signal sequence, I29, peptidase C1A |
| SnuCalCp02 | - | Signal sequence, I29, peptidase C1A |
| SnuCalCp03 | Signal sequence, I29, peptidase C1A | Signal sequence, I29, peptidase C1A |
| SnuCalCp04 | Signal sequence, I29, peptidase C1A | Signal sequence, I29, peptidase C1A |
| SnuCalCp05 | Signal sequence, I29, peptidase C1A | Signal sequence, I29, peptidase C1A |
| SnuCalCp06 | Peptidase C1A | Peptidase C1A |
| SnuCalCp07 | - | Signal sequence, I29, peptidase C1A |
| SnuCalCp08 | Signal sequence, I29, peptidase C1A | Signal sequence, I29, peptidase C1A |
| SnuCalCp09 | Signal sequence, I29, peptidase C1A | Signal sequence, I29, peptidase C1A |
| SnuCalCp10 | - | I29, peptidase C1A |
| SnuCalCp11 | Signal sequence, I29, peptidase C1A | Peptidase C1A |
| SnuCalCp12 | - | Signal sequence, I29, peptidase C1A |
| SnuCalCp13 | Peptidase C1A | Signal sequence, I29, peptidase C1A |
| SnuCalCp14 | Peptidase C1A | Signal sequence, I29, peptidase C1A |
| SnuCalCp15 | - | Signal sequence, I29, peptidase C1A |
| SnuCalCp16 | Signal sequence, I29, peptidase C1A | Peptidase C1A |
| SnuCalCp17 | Signal sequence, I29, peptidase C1A | Signal sequence, I29, peptidase C1A |
| SnuCalCp18 | Signal sequence, I29, peptidase C1A | Peptidase C1A |
| SnuCalCp19 | Signal sequence, I29, peptidase C1A | Peptidase C1A |
| SnuCalCp20 | I29, peptidase C1A | Peptidase C1A |
