# Supplementary material for: Cysteine Protease Profiles of the Medicinal Plant Calotropis procera R. Br. Revealed by De Novo Transcriptome Analysis
Source: PLoS One. 2015 Mar 18;10(3):e0119328. doi: 10.1371/journal.pone.0119328 (PMC4365007; doi:10.1371/journal.pone.0119328)
Supplement: S4 Table — (DOCX) [file pone.0119328.s005.docx]

**S4 Table. Nucleotide sequences of cysteine proteases.**

|  |  |
| --- | --- |
| **Unigene ID** | **Sequence (5’-3’)** |
| SnuCalCp01 | atgatatatgattccattatccagaaagagtttgcaaatatgaagagctttgtattaatcctttctttcttactatttgtgtcagccgtcacttgctttagtaccaattggcgaagtgatgaagaagtccttgcattatacgaggaatggctagtcaaacaccaaaaattacactccagcctaggagagaaaattaagagatttgaaatctttaaggataatcttaggtatatcgatgaacagaacaattacaacaaattcaaccacaagaattttacactcggattgaatcaatttgctgatctcacccttgatgagtttagctccatatatttaggtacgagtatcgaatatgatccaattatatcatctaatcctaaccacgacggcgaagaagaagatattcttcaagaaggtgctattgaattgccaaattctgttgattggcgaaaaaaagatgttgtttttcctattagaaatcaaggacaatgtgggagttgctggacattctcggctgttgcttcaattgaaactctaattggaattaaagaagatcgtatgattgcattatccgagcaagagctattggactgcgaaagaacaagttatgggtgtaaaggaggttactatactaatgcattcgcatatgtagccaagaaaggtcttacctctcgggaaaaatatccatatatatttcaacaaggacaatgttatcaaaaggaaaaggtggtcaaaatttctggttataggagaatacctaaaaacgatgagaaaaaacttcaaagtgttgtagcacaacaagtagtgagtgttggcgtaaaatctaaaagtagagatttccagcactatcgttcgggtgtatttagtggagcttgcggtccacgagtggatcatgcagtgaatattgttggatatggttctgaaggtggagttaattattggatcgtgagaaactcttggggtacaaattggggagagaatggatatatgagaattccaaggaattcaaatcagtctggaggttattgtggaattgctgttcaggctgcttatcctgtttattga |
| SnuCalCp02 | atggcttccttcaaactgttttcttgtttttttcttttgcttttggttgtttcgtctgcattagacatgtccattatcagttatgataatgaccatggtcagatggttaggtctgatgatgaggttaggtctttgtatgaatcttggcttgttaagcatgggaaagcttacaatgctttaggggagaaagagaaaaggtttgaaattttcaaagataatcttcagttcattgacgaacataactctaagaacctttcttacaaacttggccttaatcgtttctcggatctgagtcacgaggagtttcggtccatttttgtgagtggtcgaatggatcggaaggctaggttgatgaagggtaaggttggggatcgttattcttttaatgctggtgatgatttacccaagtctgttgattggagggagaaaggtgctgttgctcctgtcaaagatcaaggccaatgtgggagttgctgggcattctcaacagtaggtgcagttgaaggaattaataaaattgttacaggtgatttggtaaccttatcagaacaagagttggttgattgtgataatacttataaccaaggatgcaacggtggtttgatggactatgcctttgaattcataactaataatggaggtatcgatactgaggatgattacccttacaccgcccgtgatggaacatgtgatcagttcaggaaaaatgctcgagctgtttccattgatggctttgaagatgttccaaagggtgatgaacaatcattaaagaaggcagtggcccatcagccagtcagtattgccattgaagctgggggcagagcttttcagcactaccaatcgggtgtcttcactggacactgcggaacaaacctagaccacggtgttgttgctgttggatatggtactgaggatggagttgattattggttagtaagaaactcatggggtccaaaatggggagaagctggatatatcaaacttgagcgcaatgtgcacgggaacactggaaagtgtggcattgcaatggaaccttcttaccctgtgaagaacggtcctaatcctccaaatccaggtccatcccctccatccccagtaaagcctcccacacaatgtgatgattattattcatgtccggaaggaagcacctgttgctgtgtctatccgtatggtaatttctgctttggctggggatgctgtcctttggaatctgctacctgctgtgatgataacaccagctgctgcccacatgattatcccgtctgtgatatcattgaggggacttgcctaatgagtaagaacagtccaatgaagatgaaagctttgaacaggggtaatgctatgccaaactggtctcgtttgcagaggaagatcctcagttcttga |
| SnuCalCp03 | atgaagattagtttcataatattcgtatgtttctctttgctatcgatttcaaaaatcatatccattgccgatgaatccgtgtggcgaaccgaagaagaagtcatggcaatatacgaggaatggatagtgaaacacgggaaatcatacaacgcattaggagaggagaaatttaaaagattcgaaatatttaaggataatcttaaatatatcgagaaa  cacaacagtcttcccaatcaaatctacaaactcggtttgaatcaattttcggatcttacctttgacgagtttaaatccatttatttaagtagcatccctatggatacttcgttaagtgagtccaaaattgacttttctgaagttgaccttaactttcccgtgcctccttccgttgattggagagaaaaaggtgctcttgttccaattaaaaatcagggaagatgcgggagttgttgggcgttctcagcagtagcttcagtggaagccttaaataaaataaagggtggagaattaatctcattatccgaacaaatgatggtagactgtgtaaatgcgagttatggttgcaaaggaggtcgtcaaacagatgcattcaaatacatcaaagtccatggtattgcctctagtaaggactacccgtatgtaggtgtacaagggccatgtcaacccaaagaaatcgtgcttaaaattagtggttacagaggtatagttcggaataatgagaaataccttcaaattatagcatcccagcaagtagtgagtgttagcataaaagttggcaaagacttccaacactataaatccggtatatttaatggaacatgtggtgataaaattaatcatggagtgaacgttgtgggatacggatctgaaaacgggattccttattggatcgtaaggaactcttggggtaaaggatggggagaacaaggttatataagaatgcgccgaaacataagagatcctgcgggttgttgcggagttgctataacgccaacttttcctgttatcgacgaaggacatatcagcatcctttga |
| SnuCalCp04 | atggctcgtttctctctgtgtttctggtctcttgcagcagctttgaccttcttatcctttgcaatcgcttctattggagaagaatcatctaaccttataaatgaagatcctttgatcaggcaagtggtatcgggaaaggatgaggatccgcttttgaatgcagaacatcacttctcgttatttaaagctaagtttggtaagagttatgcctcactggaagagcatgattatagattatcggtgtttaaggctaatctccgccgtgctaagaggaatcagattcttgatccttctgctgtacatggcgtgactaaattttctgaccttactccttctgagttccgccggacctacctcggtcttcacaaacagaagcgccgtctctggttgcctgtcgatgcaaacaaggctcctgttctcccgactaaggatctccccactgattttgactggcgtgaccatggtgctgttactggcgtaaaagatcagggttcatgtggatcatgctggtcatttagtgcgacaggggctctagaaggtgcacattatttggcaactggtgagctagtgagcctcagtgaacagcagcttgtcgattgtgatcacgagtgtgacccagaagaaagcgggtcttgtgattctgggtgtaatggtggactgatgaacaatgccttcgaatacgtacttaaagctggtggtgttgtaagcgagcagacttatccttacacgggtactgatggaaaatgctcatttgataaaagcaaagttgttgctaaggtagccaacttcagtgttgtttcccttgatgaagatcaaattgctgcaaacttggtgaagcatggtcctcttgcagttgggatcaatgctgcttggatgcagacatatattggaggtgtttcttgcccatacatttgtggaaagcatttggatcatggagtgcttctagttgggtatggtgctgagggatatgcacctattcgcctcaaggaaaaaccttactggatcataaagaactcctggggtgaaaactggggagaggagggatactacaagatctgcaggggtcgtaacgtctgtggggtggattccatggtctcaactgtggctgctgttaatacttaa |
| SnuCalCp05 | atgaagacccttttgctgtttttctttctctctgtttctttagcttcttcggacatgtcaattattagctacgatgaaacccatggaatcagatggagaacagataatgaagtcatggacttgtacgaggcatggctagtggaacatggaaagtcatataatggtttgggtgaaaaagataagagatttgagatctttaaggataatcttagatacatagatgaacagaacagtgttgctaatcggacttataaacttggattgaatcgatttgctgaccttactaatgatgagtatcggtccatgtatttgggtacaaagactgatgctaagaggagattttctcggtctccgagcgatcggtatcttccaaaagccggtaatagcttgccggattctgttgactggagaaaggaaggtgctgtcgttccggtcaaagatcaaggcagctgcgggagttgctgggctttctctaccattgcttcagtggaaggaataagtgctgtagtaactggtgatttgatttcattgtccgagcaagagttggtagactgtgatatttcttataacgaaggatgcaacggaggtctaatggattatgcatttcagtttattattgacaatggtggtattgactctgaggaggattacccatatacggctaaagatgggagatgtgatcaatataggaaaaatgctaaggtggttaccattgatggttatgaggatgtgcctaggaataatgaggaagctcttaaaacagctgcagcaagtcaagtagtcagcgttgccattgaaggtggtggccgggacttccagctctatcgtgggggggtatttacagggaaatgtggaacctcattggatcatggtgtgaatgttgttggatatggttcagagaatggtcttgactattggatagtgaggaattcatggggtccaagctggggagagaatggctacatcaggatgcagcgtaatgtggcttcgtcttcaggactttgtggtattgccattgagccttcttacccgattaagaaaggtggaaacccaccaaatccaggtccttctcctccatctccaatcaggccacccacagtctgtgatagctattatgaatgccctgaatccaccacctgctgctgtatctttgaaatcggtaactcctgctatgaatggggttgctgtccactggaaggtgccacatgctgtgaggatcattacagttgctgcccacatgattaccctgtttgtaatgtgtatgctggcacttgttccatgagcaagaataacccacttggagttaaggtaatgaagcatatgctcgccaggcctattaagacctctggcagtgag  ggacagaagagcagctcttga |
| SnuCalCp06 | atgaatccacggttttcggatttcactgttcgccaatttaagaacctccttggggtcaaatctccacaaaggggagatttagagagcacccacattataagtcatgaaaaggggctgaaattgcccaatcagtttgatgcacgaacagcatggccacaatgtgccaccattgggagaattcttgatcagggacattgtggttcttgctgggcttttggggccgttgagactctctctgatcgtttctgcattcattttggcatgaatattagcctctctgtcaatgatctcctatcttgctgtggctttatgtgtggtaatggttgtgatggtggttatccattatccgcatggaaatactttgtctactctggtgttgtgactgaagagtgtgatccatactttgataatgaaggttgttctcatcctggttgtgaacctggatatcccactccaaagtgccatcgaaagtgtgtgaaaggaaacttgctttggaagcaggcaaagcattatagtaaaagaccacacaaaataaagtctgatccatatgatatcatggcagaagtctataaaaatgggccagttcaggtttctttcactgtctatgaggacttcgctcactaccggtcaggtgtttacaagcatattacagggagttcattggggggtcattcagtgaaattgatagggtggggaaccagtgaagatggagaagattactggctgcttgccaatcagtggaataaaagctggggtgatgaaggatacttcatgatcaaaagaggaacaaatgaatgtggcattgaagattctgtggtttcaggaatgccttcacctaaaaacttgatcaaagaactcagtagtgaggatgtgtctcttgaatcctcgagctag |
| SnuCalCp07 | atgaacatctcgtataacgaaagcagattgcctgaaattgaaggaaccatggctctccatttctcctctctgtcgttgcttctctctcttcttttagtttccacatcgtttgccttttccttatcccgtgatgatgtcagcatcgaggatactttcatccggcaggtggtcggagacaacaacgacctgttaaacgccgagcaccacttctccatctttaagaagaaattcggcaaatcttatgtttccatcgaggaggagaattacaggctttcagtgtttaaggctaatatgcgccgagcccagcgcaatcaggaacttgacccttccgctgagcacggtgtcactcaattctccgatttgactccgcgcgagttccgccgtaatgttcttggattaaagagacgatcgcgtctcagactcccttccgatgctaacaaagctccgatccttcctaccgatgatcttccttccgactttgactggagagaccgtggtgccgtcgggcctgtaaagaatcagggctcctgtggatcttgctggtcgtttagtacaactggagctttggaaggtgcaaattatttagcaactggcaagtttgtcagtctaagtgagcaacagttggtggattgtgaccacgagtgtgaccctgctgaagcagattcatgtgattcgggatgcaatggtggacttatgaacaatgcattccagtatacacttaaagctggtgggattatgcgagaggaagattatccttacactggcacagatcgtggaacttgcaaatttgatcagaccaaggttgctgccaaagttgccaactttagtgttgtctcccttgatgaagatcaaattgctgctaatcttgtcaagaatggtccacttgcagtggctattaatgcagtgtatatgcagacgtacataaaaggggtttcatgcccgtatatatgttccaaacggttagatcacggtgtgttattagtgggctatggttctgagggctatgctcccatccgtatgaaggataagccatactggattattaagaattcatggggtgaacattggggagaaaatggattctacaaaatatgtcgtggtcgcaacgtttgtggtgtggattcaatggtatctactgttgcagcagttgccacagcctccggttga |
| SnuCalCp08 | atggctcgagtatccttcgtctttctggccgtcgtagccgttctgattactgtctccgtcgccgttgacgacggatcatcagcctattttgcccaggtgaatccgatcaggcaagtcgtgtccgacggtctgcgtgaattagagaattcttttgttcaggttattggaaatactcgccatgtgctctcctttgctcgctttgctcataggtatggaaagaggtacgagactgctgaggagataaaagtgaggttcgacatattcagggacaatctgcggatgattaaatcgcataacaagaagggactgtcattcagtcttggtgttaatgcattttctgatttgacatgggaggaattccgtaagcataggttgggagctgcccagaactgttcagctaccacaaagggaaatctcaagctaaccaacgttgtcctcccagaaatgaaagactggagggagactggtatagtcagccctgtcaaagaccaaggtcactgtggatcttgctggacattcagtaccactggagccctggaggcagcttacacccaagctttcaagaaggaaatttccttgtcagagcagcagcttgtggactgtgctggagctttcaacaattttggttgccgtggtggtttgccatctcaagctttcgagtatattaagtacaatggtggacttgacacagaagaagcctatccatatgtgggaaagaatggtgtttgcaaatattcatcagaaaatgttggtgtacgagtgctagactctgtcaatattaccctgggtgctgaagatgaactcaagtatgcagtgggattgcttcggcctgttagtgtcgcattcgaggtggtaaaagatttcagacactataaaagtggtgtttacaccagcaacacatgtggcagctccccaatggatgtgaaccatgcagtccttgctgttggttatggtgttgaagacggtatcccttattggcttattaagaattcatggggtgcttcatggggtgacaatgggtacttcaaaatggagatggggaagaacatgtgcggtgtttcaacttgtgcatcattccccgtagttgcctaa |
| SnuCalCp09 | atggctgtctctactggctcgttgaaattgttgtttgctttagttactctatctttctttgccggtaaaacaatgggtcgtgaattctcaattgtgggatattcgcaagatgatttaacttgcattgacaaaatgatcaatcttttcgagtcgtggattgagaaacacggcaagatatacgaaacaattgaagaaaaattgcataggtttgagatattcaaagaaaatttgaagcatattgatgagaggaacaagattatcagtaactattggcttggtttgaatgagtttgctgatttgagtcacgaagaattcaagaacaagtttttaggacttaaggttgttggtaaagatcatcatcatcagttgtctgaaaacagacaatgttctgatgatgattttagttataagaatgttgattttgatgccattccaaaatctgttgactggagaaagaaaggtgctgttactcctgtcaagaaccaaggatcttgcggtagttgttgggcattttcaactgttgctgcagttgaaggaataaaccaaattgttactggaaatttaacttcactatcagaacaagaactcattgattgtgatactgcttacaacaatggatgcaatggaggactaatggattatgcattttctttcattgtttccaatggtggccttcacaaggaagaagattatccttacctaatggaggaaggaacttgtgatgagaaaaggactgaatcagaagttgtcaccatttctggttaccatgatgttccccagagcaatgaacaaagctttctcaaagcacttgctcaccagcccttgagcgttgccattgaagcttctggtagagatttccaattctacagtgggggtgtttttgatgggcattgtggaacagaacttgatcatggtgtggcagcagttggatatggttcaacaaaaggattggattacataattgtgaagaattcatggggacctaaatggggtgaaaaaggatacataagaatgaagagaaacactggaaaaccagaaggcatttgtggtatcaataagatggcttcttatcctactaaaactaagtga |
| SnuCalCp10 | cgaagtgatgatgaagtcattgcattatatgaggaatggctagtcaaacaccaaaaattacactccaaccttggagagaaaattaaaagatttaaaatctttaaggataatcttaggtatattgatgaacaaaataattacaataaagtcaaccacatgaactttacactcggattgaatcaatttgctgatctcacccttgatgagtttagctctatatatttaggtacgagtgtcgattatgaacaaataatatcatctaatcctaatcaccacggcgaagaagaagatatttttaaagaaggtgccgttgaattaccgaagtctgttgattggcgaaaaaaaggttttgttcttcctatcagaaatcaaaaaaaatgtggggcttgttgggcattctcggcagttgcttcaatagaaacgttaattggaattaaaaaaggtcgtacgattgcattatctgagcaagagctattggactgcgtaacagcatgtgatgggtgtaaagctggtcactatgactctgcattcgcatatgtagcgaagtatggtattacctctcgggaaaaatatccatatgtatataaaaaaggacaatgttctcgaaagaaaaaagtggtcaaaatttctaattataagagactacttagaaataatgagggacaacttcaaattgctgtagcacaacaagtagtgagtgttggagttaaagctaccagtaaagattttcagcactatcgttcgggtatatttaaaggagcttgcggtccacaattgaatcatgcagtgaatattgttggatatggttctgaaggtggagttaattattggatcgtgagaaactcttggggtacaggttggggagagaagggatatatgaggattctaaggaattcaaaacagtctgaaggttactgtggaattgccatgaagccctcttttcctgtttattga |
| SnuCalCp11 | atggttccctctccttatctccgtgttcctcttctcctagcaatggtagtagtagtattttacagccccgcggcatcagaatcaactactactaatactaatactggtgatcgtctttcaattgtgggttactcgccggaggacttgagttcagataacaggctacttgatctgttcgagtcatgggcatcgaaacacggcaaaagatacaaaagcgtggaggaaaagctgttgagatttgagaatttcatggacaatttaaagcatatcgacgaatccaataaggaggcaaataaaacttactggcttgggctgaatgaattcgcggacttgagtcacgaagagttcaggaatagatacttaggcttcagatctggacggttccccaggaggaagaggagcggagattcttctctaaatttcaattacaggaatgcctataaaataccgaaatcaatggactggagaaaaagaggagccgtcggtcatgttaaaaaccaaggtccttgtgggagttgttgggcattctcaactgttgcagctgtggaaggcataaataagatagttacgggaaattttacttcgttgtcggagcaagagctgattgactgtgacacatcttttaacaacggttgtaacggaggtctaatggactatgcattccagtacataactcaaaacggaggccttcgcaaggaggaggactacccttacctcatggatcagggcacctgcgaagcaaccaaggaagaaactgatgtagtcaccataagtggttaccaagatgtgccccaaaatgacgaacagagtctcctgaaagcacttgcgcaccagccacttagcgtcgccattgaagcttcaggccgagatttccagttctataaagggggaatattcacagggccttgtggatcacagcttgaccacggagtagcagctgtaggatacggatcatccaagggtctggattacatcattgttaggaattcttggggctctacctggggggaaagaggatacatacgaatgcagagaaacacagggaatcatgaaggactctgtggcatcaacaaaatggcttcttaccctgttaaaaagaattaa |
| SnuCalCp12 | atgaagacaagtttcataatactcctatttttctctttgctatcaatttcaaaaatcaaatccattgccgatgaattagtccggcgaactgacgaagaagtcatgtcaatatacgaggaatggatggtggaatacaggaaatcctacgacgcattaggagtggagaaattaaagagattcgaaatatttaaggataatcttaagtatatggaagagcacaacagtcttcccaatcaaacttacaagctcggtttgaaccaattttccgatcttactcttcgcgagtttaaatccatctatttaagcagcagccctattgatactttgttagatgagtccgaaattgacttttcctattttccccaagttaactataacctttctttgcctgactccgttgactggagaaaacgaggtgcagttcttccaattaaaaatcagggaagttgtgggagttgctgggcgttctcagcaatagcttcagtggaagccctaaataaaataaagggtggagatttaatctcattatcagaacaaatgctggtagactgtgtaacatccagtcacggttgcaatggaggtcgtcaaatcgatgctttccgatacatgacagaccatggtattgcctctagtgacgactacccgtatgaagctgtacgagggtcatgtaaaaacaaaaaaattgtagttaaaattaaaggttacagaagaatacttccgagaagagagagattccttctaagtagagcatccatgcaagtagtgagtgttagcataaaagctggcagtaaagacttccaacactataaatccggtatatttaatggaaaatgtggtaataaaattaatcatggagtgaacgttgtgggatacggatctgaagacgggaatgcttattggatcataaggaattcttggggtgaaaaatggggagagcaaggttatatgagaatgccacgaaatataagaaaatctgaaggttattgcggaattgctttaagaccgtcgcttcctgttatgacctctatcatatcagcatcctttgaatag |
| SnuCalCp13 | atgaagagaggaagtctgagtttaatctactcagaatgtgttgcattcgtattctgcacatttttggtctcttcttctacactccatgcaaaccctgatgaaggtctgaagatacgtcaagttacaggcaataataatgataataataataataaacttttggggacagctacggagactcatttcaagtcttttgtagaggaatacgggaaagaatactctagtcggaaggaatacgtgcaccgattgggggtttttgctaagaacctacttagagctgcagaacatcaggctatggatcccacagctgtccacggcgtaacggagttttcggatctgacggaggatgagttcgagaagatgtatatgggtgtcaaaggtgggtcccattttagttatcaacttccccgggctaatcaagattcgacgaccatgattgaagaagtaggtgacttgccggagaattttgactggcgagaccacggtgctgttactgaggtcaagacgcagggaacttgtggatcatgctgggcatttagcacaacaggtgcaatagaaggggctaactttgttgcaacaggaaagctcgtgagcctaagtgagcagcagcttgtagattgtgatcacgcgtgtgatataaaggataaagattcatgtgacgatggatgctccgggggactcatgacaaatgcttataattacttgatagaggcaggtggaatagaggaggaatcctcttatccttatactggaaaacgtggtgaatgtaagttcacacctgagaaagtagctgtaagattgcagaattttacgacggtttcaggcagtgaagaacagattgcggcccatttagttcgtaacggtccccttgcagtgggattgaatgctgtattcatgcaaacatatattggtggtgtttcgtgtccacttatttgtgggaagaaatttctaaaccatggtgttctcttggttggatatggttcgagtggtttctccataatcagattgggacacaaaccatactggattatgaagaactcatggggaaagaggtggggtgaacatggctattacagaatgtgcagaggacataacatgtgtggaatcaatacaatggtgtctgcagtggtaacccaggcttccacctga |
| SnuCalCp14 | atgagttggttatggcacttttggacggtcgttttactatttcatgtgcccatttgtttagcatcattttcatcttcttcttcttcttcttcttcgccgagtactgctgatttattcgagaattggtgtcgagaatatggaaaaacatactcttctgaacaagaaaaacagtacagacatggagtatttaaagataactatgattacattacccagcataacagtaagggtaattcgacctgtactctttcccttaacgcctttgctgatctcacccaccatgagtttaaatcccagttcttgggtctctccgcttctctcaatagtcccattcgattgaatcgaggttcctcctctgctattgggacattagatgtctttgatgattttgatattccttcttctgtcgattggagaacgaaaggagctgttacgaaagtcaaaaaccagggcagttgcggtgcatgttgggcattctcagcaacaggggcaatggaaggcatcaataagattgtaacaggatcacttatcagcttgtctgaacaagaattaattgattgtgatagatcttataacagtggctgtgaaggtggactaatggattatgcatacaagtttgttgtaaataataatggcatagatactgagaatgattaccccttccaaggtcgatctggagcttgcaatagaaacaagttgaaaagacgtgttgtgactattgatggttacagagatgtgcctgctggaaatgagaatgcacttatgaaagctgtggctgtacaacctgttagtgttggcatatgtggaagtgaaagagcatttcagttatactctgggggtgtttttactggaccatgctctacttctttagatcatgctgtattgattgtgggttatgattcacacaatggagtagattattggattgtgaagaattcatggggaacaagctggggtatagaagggtatgcttacatggcaagaaacactggcaattcagaaggggtttgtgggattaatatgttggcatcctatccagttaaaactagccccaatcctccatcaccacctcctccaggtccagttaaatgtaatattttcacctcttgctctggaggtgaaacctgctgttgctactgggaacctcttgggttttgcctttcatggaagtgctgtgagctggaatctgctgtgtgctgcaaggatcatcgccattgttgccctcaagattatcccatctgtgatacagaaagaaatctttgtctcgtgagaactggcaataccacattggttaaaccattcaagaaccaaggcctttctggagagaaaggcaagtggaattctttattccaagactggattttgtaa |
| SnuCalCp15 | atgaaaagtgttataatatttctttttttattgttattctttacaccaaaagtaatatctatcatcactactagcctccataattctcagaataaacttgtttggcgtactaatgatgaagtcatttcattatttgaggaatggttagttaaacataggaaggtatataatgctataggagaaaaagaaaagagattcgagatctttaagaataatcttaaatttattgatgagcacaatattagatatccaaacaagacttacacacttggcctaaatgtgtttgctgatcttactgatgatgagtaccaatccaagtatttaggtacccgtattcatccaaagagaaagtattttgcatctcacagtagtgatgatgatgagtatcttcacaaagttggatctgaaagcttgccggattttgttgattggagaagtaaaggtattgttcttccaattgaaaatcaaggtgattgtgggagttgttgggcattctcagcaatatgttcagtggaagggatatacgctcaaagaagtggtaaattgatatcattatctaagcaagagttggtggactgtgaaagaagaagttatggatgcgatggaggtgattattcaactgccttcgaatatattaccttctttggtgtttcttctgaaaaaagttacccatatacagctgaagatggagattgtaaatcagacatgataaaagtagttcaaattgacggttactatgatataaattataatgacgagaaagctcttcaaaagagtgtttcagaacaagtagtaagtgttgctgttaaagctaacagcgtagaattcaagctctataacagtggtatatttagtggaaaatgtggaattgaaagtgatcatgctgtgaatattattggatacggttcagaagataatgttgattattggatagtgagaaattcttggggtacagactggggagaaaatggttacatgagaatcatacgcaatacaaaagatccccaaggtcattgtggaattgccgacgaaccttcttatccagttattagaagaaaaacagaaggtggttatgagtatgaacataacactattttttga |
| SnuCalCp16 | atggctcgaatttccttgctttttttggttgtcatagtttctctgatcgccgtctcagttgcatccggtgaccgttcatcattctccgaggagaatccgatcagacaagtcgtagcggaccgcctgcgtgaactagaagcttctttccttgaagtcgtcggagacaaccgccacgtcctctcatttgctagctttgctcttaagtacggaaagaagtacgaaactgatggggagattaagaaaaggttcgaaatattcagagagaacttatggaagattgaatcccataacaagaagggactctcatatactctcggcattaatgaattttctgatatgacatgggaggaattcagcaagcgtaggttgggagcaccgcagcactgttcagcaaccaaatattcgactctaaagctcaacaacgttgctctccctaaatcgaaagactggagggatgctggtatagtcagccctgtcaaggaccaaggtaactgtggatcttgctggacatttagtaccactggagctctagaggcagcttatgcacaagcttacgggcagaatatatccctgtcagaacaacagcttctggactgtgctggagatttcaacaatttcggttgcgacggtggtttgccatctcaagccttcgagtacattaagtacagtggtggaattgaaactgaggaatcatatccttacatggaaaaggagggagaatgcaaattttattcaggaaatgtagctgttcgagttcgagattctttcaatatttcccagggcaatgaatatgacctccaagttgcagtggcatatattcgacctgtaagtgtggcattccaagtgttagaagatttcaaacagtacaaaagtggaatttacaccagcactgagtgtggaagtactccagaggatgtgaatcatgcagtcctagctgttggttttggtatagaaaacggtactccttattggattattaagaattcatggggaactgactttggcatccatggctacttcatgatggagatggggaagaacatgtgtggggttgcaacttgtgcatcattcccttttattgagtaa |
| SnuCalCp17 | atgaagaaattcgtgatattttgcttcctttctttgttaattttttcatttgcttctgagatcacgtcggttagctccgctggaaacaagaaaactaggccgccgtcgcagcgaaccgatgatgaagttgctgcattatacgaggaatggcttgtcaaacacggcaaggcgtataatgccttgggagagaaatccaatagattcgcaatatttaaggataatcttaggtatatagatgatcacaacagcaatgctaaccggacttacaaacttggtctgaaccaatttgccgatcttaccaatagtgagttccggtcaatgtatttagacacacgaatcgatgctaaaattaggaggagatcatcaaagttcgtcgtcagcgatcgatatcttcctaaagccgaagataatttgccggactctgttgactggagggaaaaaggtgctgtggttcctataaaagaccaagggagttgcgggagttgctgggctttttcagccatttctgcagttgaaggcataaatgcaatagtaactggtacattgatttcattatctgagcaagagctggtggactgcgataatagtggaggaaatgaaggttgcagtggaggtttaatggatcctgcgtttgaattcattaccgaaaatggtggtattgattccgaagaggactacccatataagggtaggcaagggacttgtgatcaatacagagtaaatgtcaaggttgttaaaattaatggttatgaagatgtacccagaaataacgagaaagcgcttaaaaaagctgtagcaagtcaagtagtgagtgctgcaatctcagctggtggtatcgacttccaattatatgagtcaggcatatattctggaagatacggcactgaattggatcacggcataagtattgtgggatacggatctgagaatggttatgattactggattctgagaaactcttggggcacaaaatggggagaagatgggtacatgaggctccgacgaaacgtgccggaatcaggaggccattgtcaaattgccacattaccttcttacccgattaagaacggaccaaatccaccaaatccaggtccttctcctccagcaccaaccaaaccgcctatcttttgcgatgatactagcgaatgcccaggcggaagcacctgctgttgtaccgctatgttctccgatatctgcttagcctggggttgctgtccgttagaagatgccgtctgctgtgaggatggatctagttgctgcccaaacgattttcccgtctgtaacttagcccaaagaacttgctccaagagcatgaagaattcccttgggcttaaggcgatgaagcacgtgctggctacgcctattattaagacctataagagcaacgatggaaagaaaaacagcttttaa |
| SnuCalCp18 | atgatggggaaggaattcctgattgctcttttgatggctgcaacctgtgttctggcaaagagtttggaagtgacagaaaaagatgtgggttccgaagaggatttatggaacttgtacgagaaatggagaagtcaccatacagtttcgagagatctaacagagaaacagaaacgttacaatgtcttcaaggctaatgcactccatgtttacaactccaacaagatggacaaaccttataagttgaaactcaacaagtttgctgatatgaccagccatgagttcagaaactactacagttctaaggtaaaacatttcaggatgctacacggaatacgagctgaaactggatttatgcacgaaaacaccagaaatcttccaacttctgttgattggagaaagcaaggagctgtaactggtgtcaaggatcaaggcagatgtggtagttgctgggcattttcaactgttgttggagtagaaggaatcaacaagatcaaaacaggacatcttatttcactgtctgagcaagaacttgtggattgtgaatcagacaatgaaggttgcaatggaggactaatggaaaatgcatatgagtttataaaaaagaaaggaggaattacaacagaacgtgcatacccttacagagcaagaaatgaactttgtgattcagaaaagatgaatgccccggtagtgaagattgatggacacgaaatggtacctgaagatgatgaagatgcattgatgaaagcagtggcaaatcagcctgtttcagttgccatagatgcatccggtataaatatgcagttctactcagagggagtatttacagggccatgcggaacagagcttgatcatggagtagcagcggtgggatacggaacaacacttgacggaacaaagtattggatagtgaagaattcatggggaacagagtggggagaaggaggatacattagaatggttcgtggtagtcatgctgaaggaggcatttgtgggatagctaaggaagcttcttatcctgtgaaattatcatcggacaatcctaagtcagtaacatccaaagatgagctctag |
| SnuCalCp19 | atggagtattattcgaagagggttttcttattagtttctctttcgttagctttgattattggaatcacagagagtttcgatttccacgaaaaggaattagaaacagaagaaagtttgtggaatttgtacgaaaggtggagaagtcatcacaccatttcaacaaatcttgatgagaaacacaggcgttttaatgtgttcagagcaaatgctcattacgttcatgaattcaacaagaaagatgacgtgccttacaagttaaagctcaacaagtttgcagacatgactaaccatgaattcagaagcttttatgccggttctaagatcaagcatcaccggacgcttaccggaagatggaacaaaacctccactttcgcgcacgctaatgttaatgctgttcctacttctattgactggagacagaaaggcgctgtcaatcctgttaaggatcaaggccagtgtggaagttgctgggcattttcaacggtagttgcagtggagggaataaactacatcaaaacaaccaaattagtttcattatcagaacaagagctagtggattgcgataatcgagaaaacgaagggtgcaatggtgggttgatggatgtagcttttgaattcatcatgaaaaatggaggaatcacaactgagcagaactatccttacagagctcgcgatggaagatgcgataaaaataaggcaagtcattcggctgtctctattgatggttatgaagatgtgcctgctaacaatgaagatgctctgcttaaagctgtggccaaccaacccgtttccgtcgccattgatgctggaggctccgatttccagttctactctgagggagtgtttacagggaagtgcgggacagaattagatcacggagtggcaatagtaggctacggaacaaccgtagacggcacaaaatattggatcgtgaggaattcttggggaccggaatggggagagaaaggatacataaggatgcaccgtggaattaatgctaaagagggactttgtggcattgcaatgcaaccctcgtatcctatcaagaactcttccactaaccctattggaaattctaaggatgaactttga |
| SnuCalCp20 | atgcatggaatgtttaaacgggaaaaattaggacaaagaacccatcagtttctagaatggaggatcgtctacgcagttcagccatgcttgtcaatttcttctgccgtaacttggctgacggcaatctcaatggctggtttggtattaaattggaagctcactacttttgctgtactgttgattcttctggcttcctgctgggcggcttctcacgcctcagctcgaaacttgaacgaattagtgtcaatggtcgaaaggcacgagcaatggatggttcaatatggacgagtgtacaaggacgacactgagaaggcaagtcgatttaagatattcaaggagaatgttgagtacatcgaatcctttaacaaagcaggcattcgttcttacaagttaggtatcaacaaatttgctgatttgactaacgaagagttcagaaaagctcgcaatggttacaaagcatcgcacttctgcagaaaagcatcaccattgtttaagtacgaaaatgtatctgcagttccagctagcatggattggagaaagaagggcgccgttacaggaatcaaggatcagggccaatgtggatgctgctgggcattttcggctgtggcagccatggaaggaattaaccaactcaccactcataagctgatctcattgtccgagcaagagctggtcgattgcgacaccagtgaagatcaaggctgcaatggtggtcttatggacgatgcatttcagttcattatctcaaacaaaggcctaaccaccgaatctaattacccttaccaaggagtcgacggaacttgcaattccaacaaggaatctaacagcgcggcaaagattaccggttacgaagatgtccctgaaaatagcgaagctgctcttctcaaggctgtggctaaccagcctgtatccgttgccatagatgctagcggttctgattttcagttctattcgagtggtgtgtttaccggcgaatgtggcacagacttggaccatggtgtcacggctgtcggttacggtaaaagcgccgaggacgggacaaagtattggctggtgaagaattcgtggggaacaagctggggagagaatggatacataagaatgcagagggatattgatgctcctgaaggtctctgtggcattgctatgcaagcttcttatccgactgcttag |
